# Knee-Related Quality of Life Compared Between 20 and 35 Years After an Anterior Cruciate Ligament Injury Treated Surgically With Primary Repair or Reconstruction, or Nonsurgically

**DOI:** 10.1177/03635465231218237

**Published:** 2024-01-17

**Authors:** Joanna Kvist, Moa Pettersson

**Affiliations:** † Unit of Physiotherapy, Department of Health, Medicine and Caring Science, Linköping University, Linköping, Sweden; ‡ Stockholm Sports Trauma Research Center, FIFA Medical Centre of Excellence, Karolinska Institute, Stockholm, Sweden; § Örebro University, Örebro, Sweden; Investigation performed at Unit of Physiotherapy, Department of Health, Medicine and Caring Science, Linko« ping University, Linko« ping, Sweden

**Keywords:** ACL injury, ACL surgery, long-term follow-up, quality of life

## Abstract

**Background:**

Quality of life (QoL) is affected up to 5 years after an anterior cruciate ligament (ACL) injury. Knee impairment and osteoarthritis (OA) development increase over time, and this may affect QoL at a long-term follow-up.

**Purpose:**

To investigate changes in health- and knee-related QoL between 20 and 35 years after ACL injury and compare it between patients treated with or without ACL surgery, as well as to study how symptomatic OA (SOA) is associated with change in QoL.

**Study Design:**

Cohort study; Level of evidence, 2.

**Methods:**

Between 1980 and 1983, 139 patients with acute ACL rupture were allocated to surgical or nonsurgical treatment of the ACL. Both groups completed a structured rehabilitation program. Of those patients, 59 were followed for 20 and 35 years after ACL rupture. After 10 crossovers, 33 patients were treated with primary repair or ACL reconstruction, and 26 were treated without ACL surgery. Combined radiographic OA and knee symptoms at 35 years was defined as SOA. QoL was assessed at 20 and 35 years after injury with the Knee injury and Osteoarthritis Outcome Score QoL (KOOS-QoL) subscale (range, 1-100), ACL-QoL questionnaire (total score and 5 subscales; range, 1-100), European QoL–5 Dimensions Questionnaire, and visual analog scale. Results were analyzed with paired and independent-sample *t* tests and chi-square tests.

**Results:**

Knee-related QoL was impaired at both 20 and 35 years after ACL injury, and differences were dependent on the measurement outcome. In the total cohort, KOOS-QoL did not change but both total ACL-QoL score (7.1 points; 95% CI, 2.2-11.9) and 4 of 5 subscales (5-10 points) decreased (*P* < .05). No differences were found between treatment groups. QoL decreased overall in patients with SOA, with a 21-point difference within-group change in KOOS-QoL (SOA or non-SOA) between 20 and 35 years of follow-up (*P* = .001; Cohen *d* = 1.0).

**Conclusion:**

An ACL injury impairs knee-related QoL for up to 35 years, with no difference between treatment approaches (initial repair or later reconstruction compared with nonsurgical treatment). The deterioration decreases with longer follow-up. Clinicians should be aware of differences in QoL depending on the measurement outcome.

An anterior cruciate ligament (ACL) injury usually leads to impairment with knee instability, muscle weakness, and lowering levels of physical activity. The injury usually also affects the patient's knee-related function continuously through life, regardless of whether the initial treatment was ACL surgery or not.^9^ Short-term follow-ups generally focus on knee function and return to sports, and recently,^3^ a large focus has also been on psychological factors, such as fear of new injury, low self-efficacy, and decreased confidence.^4,7^

Beyond functional and psychological impairments, health-related and knee-specific quality of life (QoL) is also affected in the short and long term,^3,19,26^ and higher QoL increases the odds of being satisfied with knee function.^3^ Health-related QoL can be evaluated with generic questionnaires such as the 36-item Short Form Health Survey or European Quality of Life–5 Dimensions Questionnaire (EQ-5D). A recent systematic review showed that physical components of health-related QoL measures 2 years or more after ACL injury were worse than those in noninjured individuals, whereas mental components were similar to those in a noninjured population.^19^ A previous systematic review showed that long-term QoL after ACL injury may not differ compared with a general population but is lower compared with an active population.^17^ Measures of knee-specific QoL, as measured with the QoL subscale in the Knee injury and Osteoarthritis Outcome Score (KOOS) questionnaire, ≤23 years after ACL injury or ≤16 years after ACL reconstruction (ACLR), showed poorer results for individuals with an ACL injury compared with the general population.^16,17^ Results from the national ACL register showed that health-related QoL and knee-specific QoL increase during the first years after ACLR at follow-ups for ≤5 years.^26^ In follow-ups >5 years, KOOS-QoL scores in individuals with an ACL injury are not significantly related to time since injury,^17^ and in a longitudinal cohort study, no change in QoL was found between 15 and 20 years after ACLR.^34^

The ACL-QoL questionnaire is a knee-specific questionnaire for evaluation of QoL after ACL injury. In a study on measurement properties for the ACL-QoL, the results indicated a low QoL close to injury or ACLR, which became better over time up to 25 years.^18^ Knee symptoms and radiographic osteoarthritis (OA) increase over time, which may affect QoL negatively.^19^ No studies have examined the change in QoL at follow-ups >25 years.

The treatment option after an ACL injury is rehabilitation alone or rehabilitation combined with ACLR. Treatment strategy does not seem to affect QoL, although more high-quality studies are needed.^20,36^ We have followed a cohort from initial injury in the beginning of the 1980s up to a mean of 35 years after injury and have reported data at various follow-up occasions.^1,12,13,25,28^ Patients were initially allocated to surgery with primary ACL repair, which was standard surgical treatment at that time, or nonsurgical treatment. We have previously reported on QoL 32 to 37 years after ACL injury and now want to analyze whether QoL changes from 20 to 35 years in the same patients. Thus, the aim of this study was to investigate changes in health- and knee-related QoL from 20 to 35 years after an ACL injury and compare it between patients treated with or without ACL surgery. A secondary aim was to study how symptomatic OA (SOA) at 35 years is associated with change in QoL. We hypothesized that individuals with an ACL injury would have progressively impaired knee-related QoL and that there would be no difference between ACL treatment strategy (surgery or not). We also hypothesized that having SOA at 35 years would be associated with greater deterioration in QoL 20 to 35 years after ACL injury.

## Methods

This is a prospective cohort study that followed 139 patients 20 and 35 years after their ACL injury. Patients injured their ACL between November 1980 and December 1983, were recruited consecutively, and received treatment at Linköping University Hospital, Linköping, Sweden. All patients underwent diagnostic arthroscopy at a mean of 5 days after injury. Initially, patients were allocated based on year of birth to surgical treatment (even birth year) or nonsurgical treatment (odd birth year). Most of the patients allocated to surgical treatment underwent diagnostic arthroscopy and ACL surgery the same day.^25^ The participants included in this study are part of a larger prospective cohort follow-up study.^1,12,13,25,28^

All surgical treatments were done via primary repair because this was the standard treatment for ACL ruptures in the 1980s. Surgical treatment included augmented or nonaugmented ACL repair. Primary repair of the ruptured ACL was done with multiple sutures fixed through drill holes in the lateral femoral condyle and in the anterior part of the tibia. Augmented repair was done using a strip from the iliotibial band. A few of the surgically treated patients had proximal ACL ruptures and received nonaugmented repairs. Nonaugmented repair was the standard treatment at that time for proximal ACL ruptures. However, in 1982 this was abandoned, and after 1982 all ACL repairs were augmented. Previous research from the same cohort has reported that patients without augmentation did not differ from the ones with augmentation regarding radiographic OA and consequent knee surgeries.^25^ Patients who crossed over to late ACL surgery underwent ACLR with either allograft or autograft with bone–patellar tendon–bone or iliotibial band.

Both groups completed a structured rehabilitation program focusing on strength and coordination. Patients who received surgical treatment were first immobilized in a long-leg cast for approximately 6 weeks with the knee in 30° of flexion and proceeded afterward to rehabilitation. The duration of rehabilitation was approximately 4 to 6 months, excluding the time spent in immobilization casts for those treated with surgery.^1^

At 20 and 35 years (in 2004 and 2017-2018) after initial ACL injury, patients were invited to participate in the follow-up study. At 20 years after initial injury, patients were asked to answer questionnaires on health-related QoL. At 35 years after initial injury, patients were invited to answer questionnaires on health-related QoL and have a radiographic examination of the knee as a part of a larger study.^25^ On both occasions, a letter was sent to each patient regarding the follow-up procedure and included an informed consent form, the questionnaire, and a reply-paid envelope. Up to 3 reminders were sent.

Ethics approval was granted by the regional ethics committee of Linköping (No. 2017/119-31). This article presents results from the patients who participated in both follow-ups.

One radiologist assessed OA in the tibiofemoral joint on plain weightbearing radiographs, according to the Kellgren and Lawrence scale. Kellgren and Lawrence grade 2 or higher was estimated to be radiographic OA.^37^ Patients who had undergone knee replacements by their 35-year follow-up were scored as having end-stage OA. SOA was defined as the presence of radiographic OA in the tibiofemoral joint, together with the presence of knee pain and/or symptoms defined as a ≥1-step decrease from the best response to ≥50% of items within the KOOS Pain and/or Symptoms subscales.^25^

### Outcome Measures

Knee-related QoL was evaluated by 3 self-administered questionnaires at both follow-ups: KOOS-QoL, ACL-QoL, and EQ-5D. All 3 measurements are valid for use in individuals with ACL injuries. The KOOS-QoL and ACL-QoL evaluate knee-specific QoL, and the EQ-5D evaluates generic health-related QoL.^29,33,35^ (More information about the patient-reported outcome measures can be found in the Appendix, available in the online version of this article.)

### Data Analysis

Minimal clinically important differences (MCIDs) were used to define clinically meaningful differences. The MCID for KOOS-QoL is 8 points,^2^ and for ACL-QoL it is 6 points.^27^ The Patient Acceptable Symptom State (PASS) for the KOOS-QoL subscale is 62.5 points.^30^ The EQ-5D is a generic measure, and no specific MCID for patients with an ACL injury exists. However, a critical review of previously published MCIDs for EQ-5D reported a mean MCID of 0.074 for 8 longitudinal studies that were not disease-specific.^8^ Thus, comparison with this general value should be done with the consideration that it is not specific to patients with an ACL injury.

Descriptive statistics were calculated with means and standard deviations or with median and minimum and maximum values. Categorical binary data were analyzed with the Pearson chi-square test, and continuous data were analyzed with an independent- or paired-samples *t* test, including the Levene test for equality of variances. A significance level of *P* < .05 was chosen.

## Results

### Participant Characteristics

**Figure 1.**
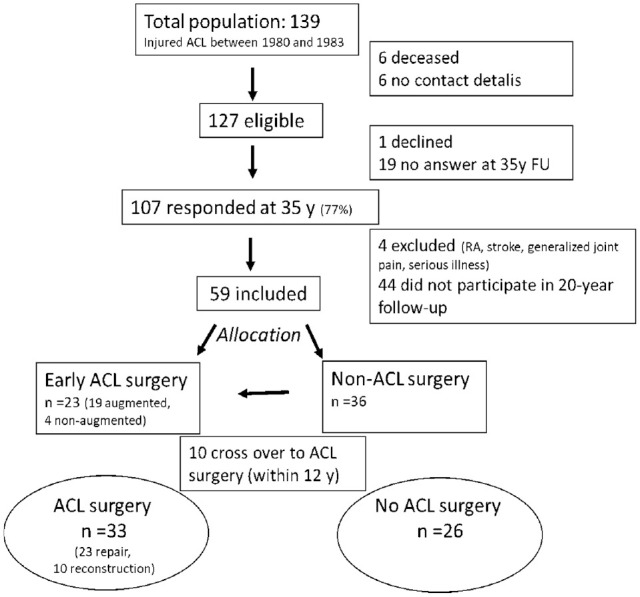
Flow of participants through the study. ACL, anterior cruciate ligament; FU, follow-up; RA, rheumatoid arthritis.

Of the 127 potential eligible patients at the 35-year follow-up, 59 patients (46%) who had answered questionnaires both times (20- and 35-year follow-ups) were included in the analyses (Figure 1).

Of the 59 patients, 23 patients were allocated to acute surgical treatment performed within 11 days from index injury. Seventeen of these had diagnostic arthroscopy on the same day as the ACL repair, and the remaining 6 patients had 3 days between diagnostic arthroscopy and ACL repair. Of the 36 patients who were allocated to nonsurgical treatment, 8 had ACLR within 3 years after the injury, and another 2 had ACLR 8 and 12 years after, respectively. Between the 20- and 35-year follow-ups, there were no new ACL surgeries. Thus, by the time of the follow-ups, 33 patients had been treated surgically and 26 had been treated nonsurgically. These are the 2 groups compared in this study. One of the surgically treated patients had gone through a knee replacement (Figure 1).

There were no statistically significant differences in patient characteristics between surgically and non–surgically treated patients. Of the 59 patients, 48 underwent radiographic examination at the final follow-up, and of these patients, 22 patients (46%) were reported to have SOA (including the 1 patient with total knee arthroplasty) (Table 1).

**Table 1:** Participant Characteristics Preinjury and at Final Follow-up (35 Years)^*a*^

	Total, N = 59	ACL Surgery, n = 33	No ACL Surgery, n = 26	*P*
Age at injury, y	21 ± 4	21 ± 4	22 ± 4	.304
Female sex	18 (31)	10 (30)	8 (31)	.969
Preinjury Tegner Activity Scale score, median (min-max)	9 (3-10)	9 (4-10)	9 (3-10)	.831
Associated meniscal injury	18 (31)	11 (33)	7 (27)	.809
Surgically treated associated meniscal injury	14 (24)	7 (21)	8 (31)	.530
Age at follow-up, y	56 ± 4	56 ± 4	57 ± 4	.631
Tegner Activity Scale score at follow-up, median (min-max)	2 (1-7)	2 (1-7)	2 (1-6)	.261
BMI at follow-up	27 ± 3	27 ± 3	27 ± 4	.648
Symptomatic osteoarthritis at follow-up^*b*^	22 (46)	14 (52)	8 (38)	.343

a Data are presented as n (%) or mean ± SD unless otherwise indicated. Comparisons between surgically and non–surgically treated patients are included. ACL, anterior cruciate ligament; BMI, body mass index; KOOS, Knee injury and Osteoarthritis Outcome Score.

b Radiographic examination of the tibiofemoral joint and evaluation of knee symptoms with KOOS Pain and Symptoms subscales. One patient with total knee arthroplasty is included. Eleven participants (19%) were missing data: 6 (18%) in the surgically treated group and 5 (19%) in the non–surgically treated group.

### Sensitivity Analysis of Nonresponders

Of the 103 patients who responded at the 35-year follow-up and were eligible for inclusion, 44 did not respond at 20 years of follow-up and were therefore excluded from the analyses. Comparisons between the responders (n = 59) and nonresponders (n = 44) at the 20-year follow-up showed no significant differences between the groups in sex (31% vs 25% female), preinjury Tegner Activity Scale score (mean, 9 [range, 3-10] vs mean 8 [range, 3-10]), or number of patients treated with ACL surgery (56% vs 68%). Nonresponders were a mean of 6 years older (mean ± SD, 21 ± 4 years vs 27 ± 7 years; *P* < .01). The 2 patients who crossed over to surgery at 8 and 12 years, respectively, reported similar patient-reported outcome measures to the entire group (no outliers).

### Quality of Life

**Figure 2.**
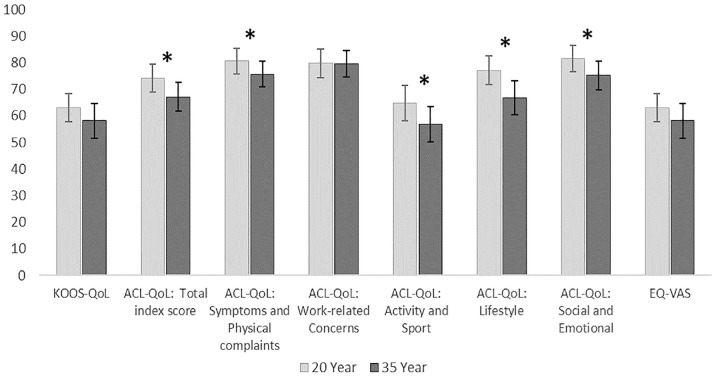
Outcome scores presented as mean score (95% CI) at 20-year and 35-year follow-ups for the total cohort. *Statistically significant changes. ACL, anterior cruciate ligament; EQ-5D, European Quality of Life–5 Dimensions Questionnaire; KOOS, Knee injury and Osteoarthritis Outcome Score; QoL, Quality of Life; VAS, visual analog scale.

Within the total cohort (N = 59), all self-reported outcomes showed a tendency to be more impaired at the 35-year follow-up than at the 20-year follow-up. Significant and clinically meaningful changes were found in the outcomes for the ACL-QoL total index score and for 3 subscales (all *P* < .05). The subscale “symptoms and physical complaints” was statistically significant (*P* = .044) but not clinically significant (within-group change of 4.9 points) from 20 to 35 years of follow-up. EQ-5D visual analog scale (EQ-5D-VAS) changed from a total mean score of 84 at the 20-year follow-up to 80 at the 35-year follow-up (*P* = .052) (Table 2, Figure 2).

**Table 2:** Quality of Life 20 and 35 Years After ACL Injury^*a*^

				Between-Group Difference	
	Total, N = 59	ACL Surgery, n = 33	No ACL Surgery, n = 26	*P*	Cohen *d*
KOOS-QoL					
20 y	63 (58 to 68)	64 (5 to 70)	63 (53 to 72)	.832	0.056
35 y	58 (52 to 65)	58 (49 to 66)	59 (48 to 70)	.852	−0.05
Within-group change	5 (–1.1 to 11.2)	6,1 (–2.1 to 14.3)	3.7 (–6.4 to 13.7)	.704	0.101
*P* value	.108	.142	.459		
Cohen *d*	0.214	0.262	0.151		
ACL-QoL: total index score					
20 y	74 (69 to 79)	74 (68 to 81)	74 (65 to 83)	.964	0.012
35 y	67 (62 to 72)	67 (60 to 74)	67 (58 to 76)	.95	−0.017
Within-group change	7.1 (2.2 to 11.9)	7.3 (1.1 to 13.5)	6.8 (–1.5 to 15)	.906	0.031
*P* value	**.005**	**.022**	.103		
Cohen *d*	0.381	0.42	0.332		
ACL-QoL: symptoms and physical complaints					
20 y	81 (76 to 85)	81 (75 to 88)	80 (71 to 88)	.71	0.101
35 y	76 (71 to 81)	78 (72 to 84)	73 (64 to 81)	.279	0.295
Within-group change	4.9 (0.1 to 9.7)	3.4 (–2.2 to 9.1)	6.9 (–1.8 to 15.7)	.477	−0.193
*P* value	**.044**	.224	.116		
Cohen *d*	0.275	0.219	0.334		
ACL-QoL: work-related concerns					
20 y	80 (74 to 85)	79 (72 to 86)	81 (72 to 91)	.682	−0.112
35 y	79 (74 to 84)	76 (69 to 83)	85 (78 to 92)	.07	−0.503
Within-group change	0.3 (–6.2 to 6.7)	3.1 (–5.4 to 11.6)	−3.8 (–14.2 to 6.6)	.296	0.287
*P* value	.937	.464	.458		
Cohen *d*	0.011	0.129	−0.157		
ACL-QoL: recreational activities and sports participation					
20 y	65 (58 to 71)	64 (56 to 73)	65 (53 to 77)	.918	−0.027
35 y	57 (50 to 63)	57 (48 to 66)	57 (46 to 68)	.975	−0.008
Within-group change	8 (1.8 to 14.2)	7.8 (–0.7 to 16.3)	8.3 (–1.5 to 18)	.939	−0.02
*P* value	**.013**	.071	.093		
Cohen *d*	0.336	0.325	0.342		
ACL-QoL: lifestyle					
20 y	77 (72 to 82)	77 (70 to 84)	77 (68 to 87)	.941	−0.019
35 y	67 (60 to 73)	66 (57 to 74)	68 (57 to 78)	.742	−0.087
Within-group change	10.4 (4.7 to 16.1)	11.2 (3.5 to 18.8)	9.4 (0.1 to 18.8)	.766	0.078
*P* value	**.001**	**.005**	**.048**		
Cohen *d*	0.472	0.519	0.408		
ACL-QoL: social and emotional aspects					
20 y	82 (76 to 87)	82 (76 to 89)	81 (72 to 90)	.837	0.054
35 y	75 (70 to 81)	75 (67 to 83)	75 (67 to 84)	.947	−0.018
Within-group change	6.4 (1.8 to 11)	7 (0.9 to 13.1)	5.6 (–2.0 to 13.1)	.759	0.081
*P* value	**.008**	**.025**	.142		
Cohen *d*	0.36	0.409	0.298		
EQ-5D index (UK index score)					
20 y	0.839 (0.805 to 0.866)	0.849 (0.811 to 0.886)	0.826 (0.762 to 0.889)	.505	0.176
35 y	0.836 (0.806 to 0.866)	0.821 (0.783 to 0.859)	0.856 (0.806 to 0.905)	.252	−0.304
Within-group change	0.002 (–0.036 to 0.041)	0.028 (–0.017 to 0.073)	−0.03 (–0.098 to 0.039)	.141	0.392
*P* value	.9	.218	.378		
Cohen *d*	0.016	0.219	−0.176		
EQ-5D-VAS					
20 y	84 (80 to 88)	87 (83 to 90)	80 (73 to 88)	.084	0.461
35 y	80 (77 to 84)	80 (75 to 85)	81 (76 to 87)	.619	−0.131
Within-group change	3.5 (0 to 7.1)	7.2 (3.1 to 11.2)	−1 (–7.2 to 5.1)	**.022**	0.62
*P* value	.052	**.001**	.731		
Cohen *d*	0.258	0.626	−0.068		

a All data are presented as mean (95% CI). Within-group change is presented as mean change from the 20-year follow-up to the 35-year follow-up; positive values represent a reduction in the mean score while negative values represent an increase. Bold *P* values indicate statistical significance. ACL, anterior cruciate ligament; EQ-5D, European Quality of Life–5 Dimensions Questionnaire; KOOS, Knee injury and Osteoarthritis Outcome Score; QoL, Quality of Life; VAS, visual analog scale.

There were no between-group (ACL surgery or non-ACL surgery) differences in change in any of the QoL outcomes, with the exception of the EQ-5D-VAS (Table 2).

Patients with SOA at 35 years of follow-up scored generally worse outcomes than patients with no SOA in all outcomes, except for the ACL-QoL domain “social and emotional aspects” and the EQ-5D-VAS. The same groups at 20 years of follow-up did not differ in the self-reported outcomes (Table 3).

Significantly and clinically meaningful differences between groups (SOA and non-SOA) were found in changes between the 20- and 35-year follow-ups for the outcomes KOOS-QoL and ACL-QoL total index score, as well as for 4 subscales (all *P* < .05). EQ-5D-VAS had a change from a total mean score of 85 at the 20-year follow-up to 78 at the 35-year follow-up (*P* = .003) (Table 3).

**Table 3:** Quality of Life 20 and 35 Years After ACL Injury by SOA^*a*^

				Between-Group Difference	
	Total, n = 47	SOA, n = 22	Non-SOA, n = 25	*P* Value	Cohen *d*
KOOS-QoL					
20 y	64 (58 to 70)	63 (55 to 70)	65 (56 to 74)	.632	−0.141
35 y	59 (51 to 66)	46 (35 to 57)	70 (61 to 78)	**.001**	−1.042
Within-group change	5.3 (−1.6 to 12.1)	16.5 (5.3 to 27.6)	−4.6 (−11.5 to 2.3)	**.001**	1.000
*P* value	.129	**.006**	.183		
Cohen *d*	0.226	0.656	−0.274		
ACL-QoL: total index score					
20 y	74 (68 to 80)	70 (61 to 80)	77 (70 to 84)	.243	−0.343
35 y	67 (62 to 73)	59 (50 to 68)	74 (67 to 82)	**.006**	−0.828
Within-group change	6.5 (1.5 to 11.5)	11.2 (2.9 to 19.4)	2.5 (−3.7 to 8.8)	.085	0.511
*P* value	**.012**	**.010**	.407		
Cohen d	0.376	0.601	0.165		
ACL-QoL: symptoms and physical complaints					
20 y	81 (75 to 86)	78 (69 to 87)	83 (76 to 90)	.348	−0.284
35 y	75 (70 to 81)	67 (60 to 75)	82 (75 to 89)	**.005**	−0.876
Within-group change	5.5 (0.5 to 10.4)	10.7 (2.0 to 19.3)	0.9 (−4.5 to 6.3)	**.047**	0.610
*P* value	**.032**	**.018**	.729		
Cohen *d*	0.330	0.562	0.072		
ACL-QoL: work-related concerns					
20 y	80 (74 to 86)	74 (63 to 85)	86 (79 to 92)	.049	−0.602
35 y	79 (74 to 85)	70 (61 to 79)	87 (81 to 93)	**.002**	−0.993
Within-group change	1.1 (−5.9 to 8.1)	4.2 (−7.9 to 16.4)	−1.3 (−10.2 to 7.5)	.435	0.234
*P* value	.760	.477	.756		
Cohen *d*	0.045	0.162	−0.062		
ACL-QoL: recreational activities and sports participation					
20 y	64 (56 to 71)	62 (50 to 73)	66 (56 to 76)	.583	−0.16
35 y	57 (49 to 64)	48 (38 to 59)	64 (54 to 73)	**.026**	−0.668
Within-group change	7.3 (0.3 to 14.2)	13.5 (2.3 to 24.8)	1.9 (−6.9 to 10.8)	.096	0.493
*P* value	**.042**	**.021**	.655		
Cohen *d*	0.302	0.533	0.089		
ACL-QoL: lifestyle					
20 y	77 (71 to 83)	72 (62 to 82)	81 (74 to 88)	.148	−0.426
35 y	67 (60 to 74)	57 (46 to 67)	76 (68 to 85)	**.004**	−0.879
Within-group change	9.4 (3.6 to 15.1)	15.2 (7.2 to 23.2)	4.4 (−3.8 to 12.6)	.059	0.561
*P* value	**.002**	**.001**	.276		
Cohen *d*	0.473	0.845	0.218		
ACL-QoL: social and emotional aspects					
20 y	81 (76 to 87)	79 (69 to 88)	84 (77 to 91)	.355	−0.271
35 y	76 (70 to 82)	71 (61 to 81)	80 (73 to 87)	.119	−0.46
Within-group change	5.7 (1.1 to 10.3)	7.8 (1.0 to 14.7)	4.0 (−2.6 to 10.6)	.404	0.244
*P* value	**.016**	**.026**	.228		
Cohen d	0.362	0.509	0.242		
EQ-5D index (UK index score)					
20 y	0.85 (0.817 to 0.883)	0.832 (0.78 to 0.884)	0.866 (0.82 to 0.911)	.314	−0.295
35 y	0.837 (0.803 to 0.871)	0.771 (0.733–0.81)	0.893 (0.848 to 0.937)	**<.001**	−1.208
Within-group change	0.013 (−0.028 to 0.054)	0.061 (−0.004 to 0.125)	−0.027 (−0.079 to 0.025)	.032	0.639
*P* value	.529	.066	.294		
Cohen *d*	0.092	0.414	−0.210		
EQ-5D-VAS					
20 y	85 (81 to 88)	85 (81 to 90)	84 (79 to 90)	.768	0.086
35 y	80 (76 to 84)	78 (72 to 84)	81 (77 to 86)	.382	−0.256
Within-group change	5.0 (0.9 to 9.1)	7.3 (2.8 to 11.9)	3.0 (−3.8 to 9.7)	.293	0.308
*P* value	**.019**	**.003**	.374		
Cohen *d*	0.351	0.710	0.178		

a All data are presented as mean (95% CI). Within-group change is presented as mean change from the 20-year follow-up to the 35-year follow-up; positive values represent a reduction in the mean score while negative values represent an increase. Bold *P* values indicate statistical significance. ACL, anterior cruciate ligament; EQ-5D, European Quality of Life–5 Dimensions Questionnaire; KOOS, Knee injury and Osteoarthritis Outcome Score; QoL, Quality of Life; SOA, symptomatic osteoarthritis; VAS, visual analog scale.

### QoL Compared With PASS

The mean KOOS-QoL for both the total cohort and the surgically and non–surgically treated ACL groups was just above the PASS score at 20 years of follow-up and was below the PASS at 35 years of follow-up (Table 2). Patients without SOA at 35 years of follow-up scored KOOS-QoL above the PASS, whereas patients with SOA scored a mean of 16.5 points below the PASS (Table 3).

## Discussion

Knee-related QoL was impaired at both 20 and 35 years after ACL injury. However, the deterioration was small during the last 15 years, with clinically and statistically significant differences depending on the measurement outcome. QoL measured with the KOOS-QoL subscale or with the EQ-5D did not show any significant deterioration compared with the ACL-QoL, with clinically significant deteriorations in the total score and 3 of the 5 subscales (“recreational activities and sports participation,”“lifestyle,” and “social and emotional aspects”). Treatment approach (ACL surgery or not) did not significantly influence the long-term outcomes. QoL was decreased overall in patients with SOA with significantly and clinically meaningful differences compared with patients without SOA.

In accordance with previous studies with >5 years of follow-up,^17^ QoL did not change over time when measured with the knee-specific KOOS-QoL. The KOOS-QoL subscale is the most affected KOOS subscale during the first years after an ACL injury and ACLR.^6,26^ Our participants scored lower compared with population-based reference data for the KOOS ^32^ and were just above the PASS threshold for the KOOS-QoL^30^ by 20 years after ACL injury. The KOOS-QoL contains only 4 questions and may not reflect the entire construct of QoL. The questions are about awareness of the knee problems, modifications of lifestyle, lack of confidence, and general difficulties with the knee. All these aspects may be highly affected close after the injury, but awareness of the knee and lack of confidence may resolve with time.^14,24,31^ Modification and adaptation of activities is commonly recommended after ACL injury, especially if the injury is treated without reconstruction.^9^ That is generally not experienced as negative after reprioritizing activities,^17,31^ and patients may choose to modify their activity level to have a good knee function without ACL surgery.^21^ We did not find differences in KOOS-QoL between patients treated with or without surgery, although patients with SOA at 35 years of follow-up scored worse in KOOS-QoL (24 points lower; effect size, 1.0) than patients without SOA. Thus, The KOOS-QoL may be more sensitive for OA than other aspects after ACL injury.

The ACL-QoL is a specific questionnaire with 31 items in 5 domains and may be a better description of the construct of QoL.^29^ In addition, in the present study, absolute score numbers in the ACL-QoL questionnaire show that QoL was not affected to the same degree as measures with the KOOS-QoL, which could be interpreted as the ACL-QoL being more sensitive to detecting changes. The largest change in score between the 2 follow-up occasions was seen in the subscale regarding patients’ lifestyle. Aspects regarding lifestyle could be the effect their knee function has on general safety issues, or reduction in their enjoyment of life and everyday activities. Increased concerns regarding lifestyle after an ACL injury, for example, often being aware of their knee impairments and having to adapt their lifestyle because of it, have been reported earlier as negative experiences related to ACL injury.^24,31^

The greatest impairment at both 20 and 35 years of follow-up with a significant decrease over the last 15 years was in the subscale “recreational activities and sports participation,” in accordance with previous studies.^15,34^ It is well known that most ACL injuries occur in active athletes with high expectations regarding their ability to participate in sport.^4,14,38^ The majority of our patients participated in competitive sports before their injury, as indicated from the high preinjury Tegner activity score. At the final follow-up, participation had decreased to activities of daily living and sedentary work (Tegner scores 2-7). This may not be so surprising given the participants’ mean age at the final follow-up (56 ± 4 years). However, in a previous follow-up including 100 patients from the initial study cohort, the patients had decreased their activity level at 15 years after injury (Tegner score 5 or 6), indicating that activity level continuously decreased since the time of injury.^28^ A systematic review has shown that only 55% return to their sports after the injury,^5^ which, together with the physical impairments caused by an ACL injury, can be one explanation as to the extensive effect the injury has on QoL. Returning to sport has, in previous research, been shown to have an effect on knee-related QoL and is associated with higher KOOS-QoL and ACL-QoL scores.^4,15^ At the same time, returning to sport can increase the risk of reinjury and, for that reason, can instead have a negative effect.^11^

The scores for the “work-related concerns” subscale in the ACL-QoL were not statistically different between the 20-year and 35-year follow-ups. After an ACL injury, patients usually adapt their activities because of their knee impairments and may choose to change their plans for future work.^10,31^ Thus, a potential explanation for the minimal effect on work is that the patients’ work tasks are suitable for their capability and are not affected by their knee impairments. Likewise, patients may avoid doing activities they know cause symptoms and physical complaints and are therefore not that troubled by them. This activity avoidance may have an effect on QoL,^15,24^ as has been shown in the other subscales of the ACL-QoL.

The results from the generic measure of QoL (EQ-5D) showed no difference between the 2 follow-ups, in accordance with previous studies.^16,17^ However, when comparing our results with the reference values for people aged 35 to 44 years for Swedish and UK populations, our patients had more impaired QoL 20 years after the ACL injury. On the contrary, at the final follow-up, 35 years after injury, our patients scored higher compared with the reference values in the same age group (55-64 years).^22^ Thus, it appears that after an ACL injury, patients become somewhat more impaired regarding their health-related QoL earlier in their life than the normal population but do not become impaired to the same extent as the reference population at the long-term follow-up. The reason for that needs to be further explored.

Treatment strategy—surgical or nonsurgical treatment of the ACL—did not affect reported QoL, in line with previous studies.^17,20^ One long-term consequence of ACL injury is OA, and in the present study, 46% had SOA. There was no difference in SOA between the surgically and non–surgically treated patients, in line with our previously reported results at 35 years of follow-up of the larger cohort.^25^ The presence of OA after an ACL injury has been reported to have an additional negative effect not only on knee-related impairments but also on QoL in general.^23^ Accordingly, our patients with SOA reported worse QoL, and the largest difference (highest effect size) in our subgroup analyses was in KOOS-QoL between patients with or without SOA.

This study is one of very few prospective cohort studies with follow-up >30 years after ACL injury, with data from consistent follow-ups for the same studied population. It is also one of few comparing surgically or non–surgically treated individuals after ACL injury.

The study also has some limitations. Our patients received treatment for the ACL when primary repair was the standard surgical treatment. Since then, the surgical techniques for ACL treatment have evolved. In addition, rehabilitation protocols have also evolved since our patients received treatment. Therefore, our results may not reflect long-term outcomes for patients treated today. Another limitation is that 10 patients initially allocated to having nonsurgical treatment crossed over to have ACL surgery. Two of them crossed over at 8 and 12 years after injury, respectively. There are too few patients to make sensitivity analyses, although these patients did report similar results compared with the rest of the patients in the surgically treated group. In addition, the number of participants in the study decreased after excluding patients who did not respond to the 20-year follow-up, raising the possibility of selection bias.

## Conclusion

An ACL injury impairs knee-related QoL for up to 35 years, with no difference between treatment approach (initial repair or later reconstruction compared with nonsurgical treatment). The deterioration is small over the last years. Clinicians should be aware of differences in QoL depending on the measurement outcome.

## Supplemental Material

sj-pdf-1-ajs-10.1177_03635465231218237 – Supplemental material for Knee-Related Quality of Life Compared Between 20 and 35 Years After an Anterior Cruciate Ligament Injury Treated Surgically With Primary Repair or Reconstruction, or Nonsurgically Click here for additional data file. Supplemental material, sj-pdf-1-ajs-10.1177_03635465231218237 for Knee-Related Quality of Life Compared Between 20 and 35 Years After an Anterior Cruciate Ligament Injury Treated Surgically With Primary Repair or Reconstruction, or Nonsurgically by Joanna Kvist and Moa Pettersson in The American Journal of Sports Medicine
