# Supplementary material for: Knee-Related Quality of Life Compared Between 20 and 35 Years After an Anterior Cruciate Ligament Injury Treated Surgically With Primary Repair or Reconstruction, or Nonsurgically
Source: Am J Sports Med. 2024 Jan 17;52(2):311–9. doi: 10.1177/03635465231218237 (PMC10838479; doi:10.1177/03635465231218237)
Supplement: sj-pdf-1-ajs-10.1177_03635465231218237 – Supplemental material for Knee-Related Quality of Life Compared Between 20 and 35 Years After an Anterior Cruciate Ligament Injury Treated Surgically With Primary Repair or Reconstruction, or Nonsurgically [file sj-pdf-1-ajs-10.1177_03635465231218237.pdf]

Knee-Related Quality of Life Compared between 20 and 35 Years After an Anterior Cruciate Ligament Injury Treated Surgically with Primary Repair or Reconstruction, or Non-Surgically

Joanna Kvist, PhD[1,2], Moa Pettersson, MD-student[3]

[1] Unit of Physiotherapy, Department of Health, Medicine and Caring Science, Linköping University, Sweden
[2] Stockholm Sports Trauma Research Center, FIFA Medical Centre of Excellence, Karolinska Institute, Sweden
[3] Örebro University, Sweden

APPENDIX

Patient reported outcomes used in the study

KOOS-QoL. KOOS-QoL is a subscale from the Knee injury and Osteoarthritis Outcome Score (KOOS) questionnaire. KOOS is a validated knee-specific measurement that can be used for young, middle-aged, and elderly patients suffering knee injury and/or knee osteoarthritis.[5] This questionnaire consists of 5 subscales: pain, symptoms, activities of daily living, sports, and recreation function and knee-related quality of life. Each subscale is scored separately from a scale of 0–100, where a high score indicates a more benign outcome. For this study, only the Quality of Life subscale (KOOS-QoL), containing 4 items, was used as an outcome measurement.[5]

ACL-QoL. Anterior Cruciate Ligament Quality of Life questionnaire (ACL-QoL) is a patient-reported measurement that evaluates QoL specifically for individuals with ACL injuries.[1,3] This measurement was developed to more correctly assess how ACL injuries affects patients' QoL over the long term, and consists of 31 question on 5 subscales: "*symptoms and physical complaints*", "*work-related concerns*", "*recreational activities and sports participation*", "*lifestyle*" and "*social and emotional aspects*". ACL-QoL is scored from 0 to 100, where the latter is the best possible outcome.[1,3]

EQ-5D. The European Quality of Life-5 Dimensions Questionnaire (EQ-5D) is a generic non-disease–specific instrument assessing health-related QoL developed by EuroQoL Group, an international research group established in 1987.[2,4] The questionnaire includes 5 dimensions; mobility, self-care, usual activities, pain/discomfort, and anxiety/depression. Scores from the domains are converted to a single summary index, the EQ-5D index, and range between –0.594 and 1, where 0 equals one's health status as worse than being dead and 1 equals a complete health status. In addition to the EQ-5D index, the EQ-5D includes a visual analogue scale (VAS) that enables patients to self-rate their current health state. The scale is scored from 0 to 100, where the best imaginable health state is scored as 100 and worst imaginable health state is scored as 0.[2,4]

References

1. Filbay SR, HT Grevnerts, S Sonesson, H Hedevik, J Kvist. The Swedish version of the Anterior Cruciate Ligament Quality Of Life measure (ACL-QOL): translation and measurement properties. Qual Life Res, 2023; 32(2): 593-604.
2. Janssen MF, A Szende, J Cabases, JM Ramos-Goñi, G Vilagut, HH König. Population norms for the EQ-5D-3L: a cross-country analysis of population surveys for 20 countries. Eur J Health Econ, 2019; 20(2): 205-216.
3. Mohtadi N. Development and validation of the quality of life outcome measure (questionnaire) for chronic anterior cruciate ligament deficiency. Am J Sports Med, 1998; 26(3): 350-9.
4. Rabin R, F de Charro. EQ-5D: a measure of health status from the EuroQol Group. Ann Med, 2001; 33(5): 337-43.
5. Roos EM, HP Roos, C Ekdahl, LS Lohmander. Knee injury and Osteoarthritis Outcome Score (KOOS)--validation of a Swedish version. Scand J Med Sci Sports, 1998; 8(6): 439-48.
